# An increase in mitochondrial TOM activates apoptosis to drive retinal neurodegeneration

**DOI:** 10.1038/s41598-022-23280-z

**Published:** 2022-12-14

**Authors:** Agalya Periasamy, Naomi Mitchell, Olga Zaytseva, Arjun S. Chahal, Jiamin Zhao, Peter M. Colman, Leonie M. Quinn, Jacqueline M. Gulbis

**Affiliations:** 1 Structural Biology Division, The Walter and Eliza Hall Institute of Medical Research, Parkville, VIC 3052 Australia; 2 Department of Medical Biology, The University of Melbourne, Parkville, VIC 3052 Australia; 3 Department of Cancer Biology and Therapeutics, John Curtin School of Medical Research, Australian National University, Canberra, ACT Australia

**Keywords:** Mitochondria, Cell death

## Abstract

Intronic polymorphic *TOMM40* variants increasing *TOMM40* mRNA expression are strongly correlated to late onset Alzheimer’s Disease. The gene product, hTomm40, encoded in the *APOE* gene cluster, is a core component of TOM, the translocase that imports nascent proteins across the mitochondrial outer membrane. We used *Drosophila melanogaster* eyes as an in vivo model to investigate the relationship between elevated Tom40 (the *Drosophila* homologue of hTomm40) expression and neurodegeneration. Here we provide evidence that an overabundance of Tom40 in mitochondria invokes caspase-dependent cell death in a dose-dependent manner, leading to degeneration of the primarily neuronal eye tissue. Degeneration is contingent on the availability of co-assembling TOM components, indicating that an increase in assembled TOM is the factor that triggers apoptosis and degeneration in a neural setting. Eye death is not contingent on inner membrane translocase components, suggesting it is unlikely to be a direct consequence of impaired import. Another effect of heightened Tom40 expression is upregulation and co-association of a mitochondrial oxidative stress biomarker, *Dm*Hsp22, implicated in extension of lifespan, providing new insight into the balance between cell survival and death. Activation of regulated death pathways, culminating in eye degeneration, suggests a possible causal route from *TOMM40* polymorphisms to neurodegenerative disease.

## Introduction

The protein translocase of the outer mitochondrial membrane (TOM) is a heteromeric assembly comprising a core of Tom40, Tom22, Tom5, Tom6, Tom7 and associated receptor components Tom20 and Tom70. TOM subunits span the outer membrane with surfaces in the cytosol and intermembrane space. In humans, TOM is responsible for post-translational transfer of over 1100^1^ different nascent precursor proteins from cytosolic ribosomes to mitochondria^2^. During import, tail-anchored Tom20 and Tom22 receptors facing the cytosol accept protein precursors^3^ and transfer them to the embedded pore, Tom40, which in turn transfers them to collaborating mitochondrial translocases in the inner and outer mitochondrial membranes for sorting and assembly^4,5^. TOM components have also been implicated in mediating aspects of quality control, including PINK1-Parkin mitophagy^6^ autophagy^7^ and endoplasmic reticulum (ER)-mitochondria communication^8^, and in ageing^9^ and the pathogenesis of Parkinson’s disease^10^.

The human Tom40 (hTomm40) gene (*TOMM40*) is in linkage disequilibrium with the apolipoprotein E (*APOE*) gene on chromosome 19^11^. The ε4 allele of the apolipoprotein gene has long been recognised as a potent genetic risk factor and the strongest predictor of late onset Alzheimer’s disease^12,13^. Because of this, strong correlations between nucleotide polymorphisms of *TOMM40* and Alzheimer’s disease have commonly been attributed to the transposition of *APOE/TOMM40* regulatory elements^14^. However, this does not explain why *APOE* and *TOMM40* are independently linked to age of onset distributions for Alzheimer’s disease^15^, and why in individuals homozygous for *APOE* ε3 (*i.e.* null for the ε4 allele), distinct *TOMM40* variants differentially increase disease risk and age of onset^16,17^. There is a growing appreciation that upregulated *TOMM40* gene expression due to *TOMM40* polymorphisms is sufficient to predispose individuals to Alzheimer’s disease^16,18–21^, and an emerging school of thought endorses *TOMM40* as an independent genetic determinant of Alzheimer’s pathology^22,23^.

Despite the weight of genetic evidence linking *TOMM40 *dysregulation to Alzheimer’s Disease, the cellular pathways responsible remain unclear. Here we used the *Drosophila melanogaster* eye to model the relationship between elevated Tom40 expression and neurodegeneration in vivo.

## Results

### Elevated Tom40 causes dose-dependent retinal degeneration

**Figure 1:**
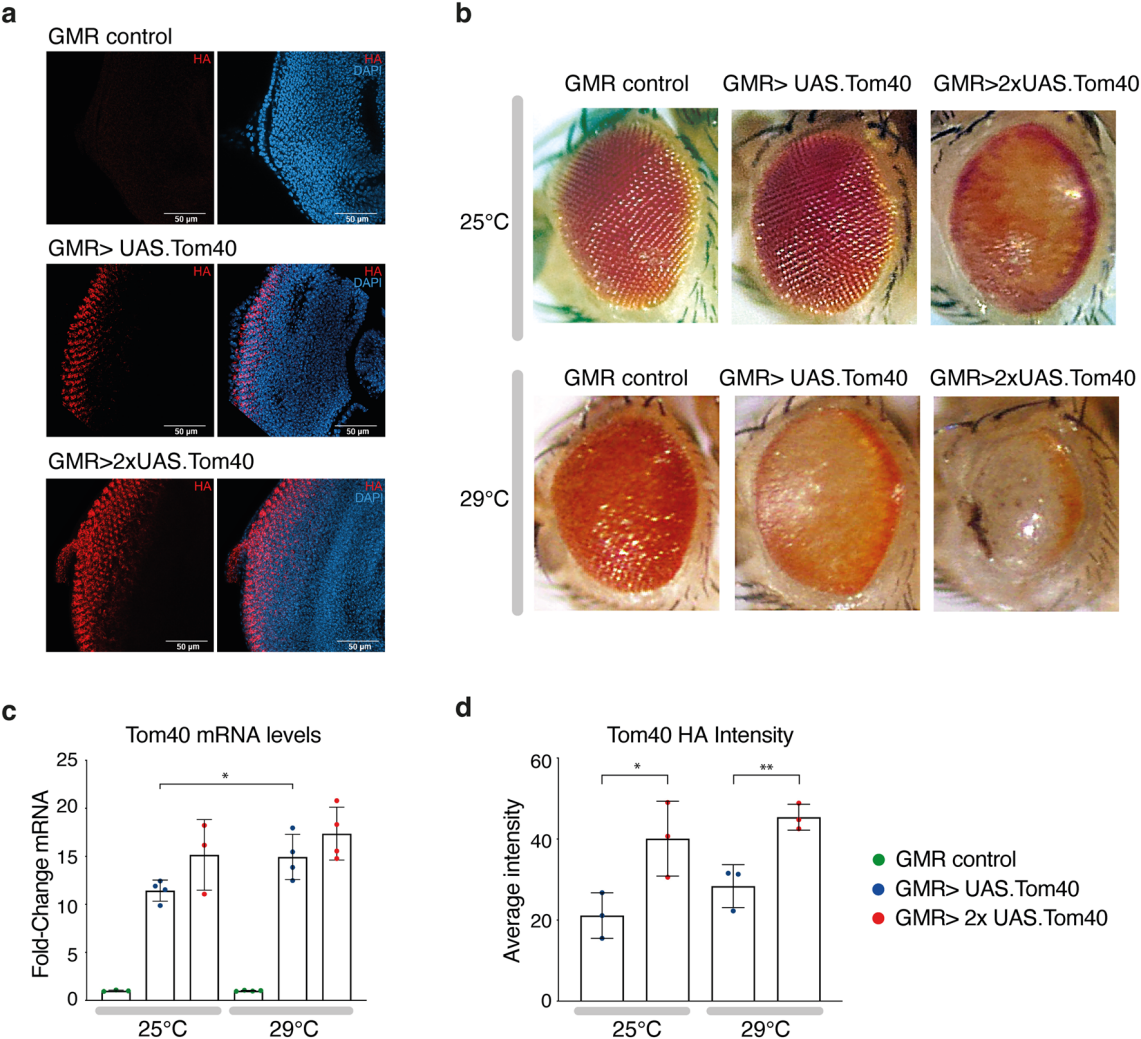
Tom40 overexpression causes eye degeneration. (**a**) Confocal microscopy of immunofluorescence staining for Tom40 overexpression in imaginal eye discs of third instar *Drosophila* larvae (control GMR-GAL4, single-copy *UAS.Tom40* and double-copy *UAS.Tom40*) stained with HA antibody (red) against the epitope tag and DAPI (blue) for DNA/nuclei. (**b**) Representative images of adult eyes for genotypes as labelled. Rearing temperature is as indicated. For each genotype, 8–10 flies were imaged using light microscopy. (**c**) Quantification of total Tom40 abundance in *Drosophila* third instar larval eye discs at 25 °C and 29 °C with 1 or 2 copies of the Tom40 gene, as assessed by qPCR, normalised to a driver-only control. Data are represented as mean ± SD from three or four independent experiments (p value = 0.0359 by unpaired t-test). The colour coding is the same in panels (**c**) and (**d**). Asterisks indicate p-values ≤ 0.05 by t-test. (**d**) Relative Tom40 expression based on intensity of HA staining (n = 3 for each column from independent eye discs) * denotes p = 0.0384, ** denotes p = 0.0089 using unpaired t-test).

*Drosophila melanogaster* eyes are rich in photoreceptors (differentiated neurons specialised for phototransduction) and have been used widely as a model for in vivo studies of neurodegenerative diseases^24,25^. To explore potential connections between elevated Tom40 expression and neurodegeneration, we expressed C-terminally-FLAG.HA-tagged Tom40 in *Drosophila* eye cells using the UAS-GAL4 system^26^, which enables tissue-specific modulation of gene expression. We used the Glass Multiple Reporter (GMR) promoter to drive GAL4 expression throughout larval and adult development in the differentiated domain of the eye^27^. The eye differentiates to generate an organized array of photoreceptor cells containing densely packed mitochondria to support the high energy requirements of phototransduction. Immunofluorescence and confocal microscopy of third instar larval eye imaginal discs (the precursors to adult eyes) overexpressing Tom40, verified expression in differentiated cells at the posterior of the imaginal discs (Fig. 1a).

Fly eyes expressing one copy of *UAS.Tom40* reared at 25 °C have normal eyes, whereas two copies cause moderate disruption to ommatidial structure and some loss of pigmentation, suggesting a dose-dependent effect of Tom40 expression on phenotype (Fig. 1b). Phenotypes driven by GMR-GAL4 are more severe in flies reared at a higher temperature of 29 °C^28^. Indeed, flies expressing one copy of the *UAS.Tom40* transgene reared at 29 °C display a phenotype comparable to those expressing two copies of Tom40 raised at 25 °C (Fig. 1b), while flies expressing two copies of the transgene at 29 °C show severe reduction of eye tissue, white glassy eyes with dark spots indicative of necrosis, disruption of ommatidial structure indicating neural degeneration, and lack of red pigmentation indicating strong induction of pigment cell death^29^ (Fig. 1b).

Tom40 expression in the posterior of larval eye discs under these conditions was quantified by complementary methods: comparative fluorometric intensity and qPCR. Adult flies carrying one copy of the Tom40 transgene, driven by GMR-GAL4 and raised at a standard growth temperature of 25 °C, exhibit a normal eye phenotype but Tom40 mRNA is increased 10- to 13-fold fold relative to a driver-only (GMR) control (Fig. 1c). We observed a significant increase in fluorescent Tom40-HA staining in eye ommatidia expressing two copies of the Tom40 transgene over those carrying one copy (Fig. 1d and Suppl. Fig. S1) and measured a significant increase in Tom40 mRNA in larvae raised at 29 °C (Fig. 1c). Importantly, comparable Tom40 mRNA levels were recorded in cohorts of larvae raised at 29 °C carrying 1x*UAS.Tom40* and those raised at 25 °C carrying 2x*UAS.Tom40*. Both methods indicate a similar upward trend in Tom40 expression with increasing temperature or additional copies of the ectopic gene.

### Elevated Tom40 activates caspase-dependent cell death

**Figure 2:**
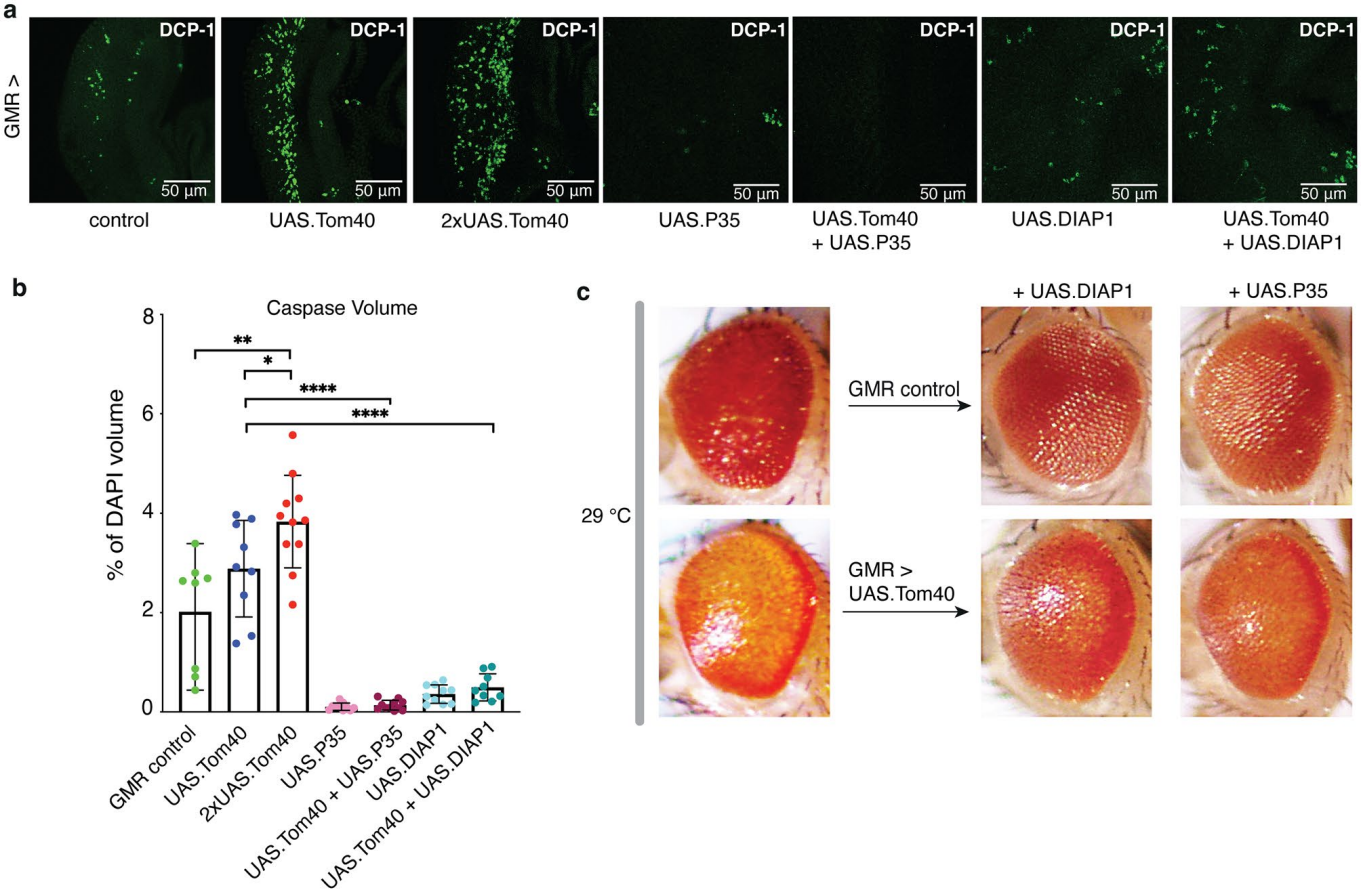
Tom40 overexpression activates caspase-dependent cell death. (**a**) Representative larval eye disc images stained with anti-cleaved DCP-1 (green), from left to right: control GMR-GAL4 (n = 8), single-copy *UAS.Tom40* (n = 9) and double-copy *UAS.Tom40* (n = 11), *UAS.P35* (n = 10), *UAS.P35* + *UAS.Tom40* (n = 11), *UAS.DIAP1* (n = 10), and *UAS.DIAP1* + *UAS.Tom40* (n = 9). (**b**) Quantification of cleaved DCP-1 proportional to tissue volume (determined using DAPI) mean volume ± SD (genotypes as labelled). ** denotes p = 0.0014, * denotes p = 0.0396, ****(upper) denotes p < 0.0001, ****(lower) denotes p = 0.0017, using unpaired t-test (< 0.05). (**c**) Representative images of adult eyes for genotypes as labelled. Rearing temperature as indicated. For each genotype, 8–10 flies were imaged using light microscopy.

Mechanisms of caspase-dependent cell death are conserved from humans to flies^28–30^. To investigate whether the tissue death associated with Tom40 expression was caused by apoptosis, we analysed larval eye imaginal discs using an antibody against activated (cleaved) *Drosophila* caspase-1 (DCP-1), an executioner caspase with a high degree of homology to human caspase-3^31^. Relative to the driver-only control, elevated DCP-1 staining was detected in the posterior of the imaginal discs of larvae expressing one copy of the Tom40 transgene (Fig. 2a), with a significant increase in DCP-1 positive cells in larval eyes expressing two copies of Tom40 (Fig. 2b). Moreover, eye degeneration was suppressed by co-expression of Tom40 with the death-associated inhibitor of apoptosis, DIAP1, an inhibitor of the initiator caspase Dronc^32^, and by the baculoviral caspase inhibitor P35, which prevents apoptosis downstream of the executioner caspases *Dm*Caspase-1 (DCP-1) and DrICE^33^ (Fig. 2c), accompanied by a decrease in DCP-1 positive cells in both instances (Fig. 2a,b). Thus, upregulation of Tom40 expression in the differentiating photoreceptor cells of the eye increases caspase-dependent cell death and severity of degeneration in a dose-dependent fashion, validating apoptosis as the underlying cause of eye ablation. Apoptosis does not proceed by the intrinsic mitochondrial pathway of *Drosophila*, however, as neither co-expression of the anti-apoptotic Bcl-2 protein Buffy (or its RNAi), or the pro-apoptotic protein Debcl (or its RNAi), significantly alter the Tom40-linked phenotype (Suppl. Fig. S2).

### Tom40 localises to mitochondria and integrates into high-order assemblies

**Figure 3:**
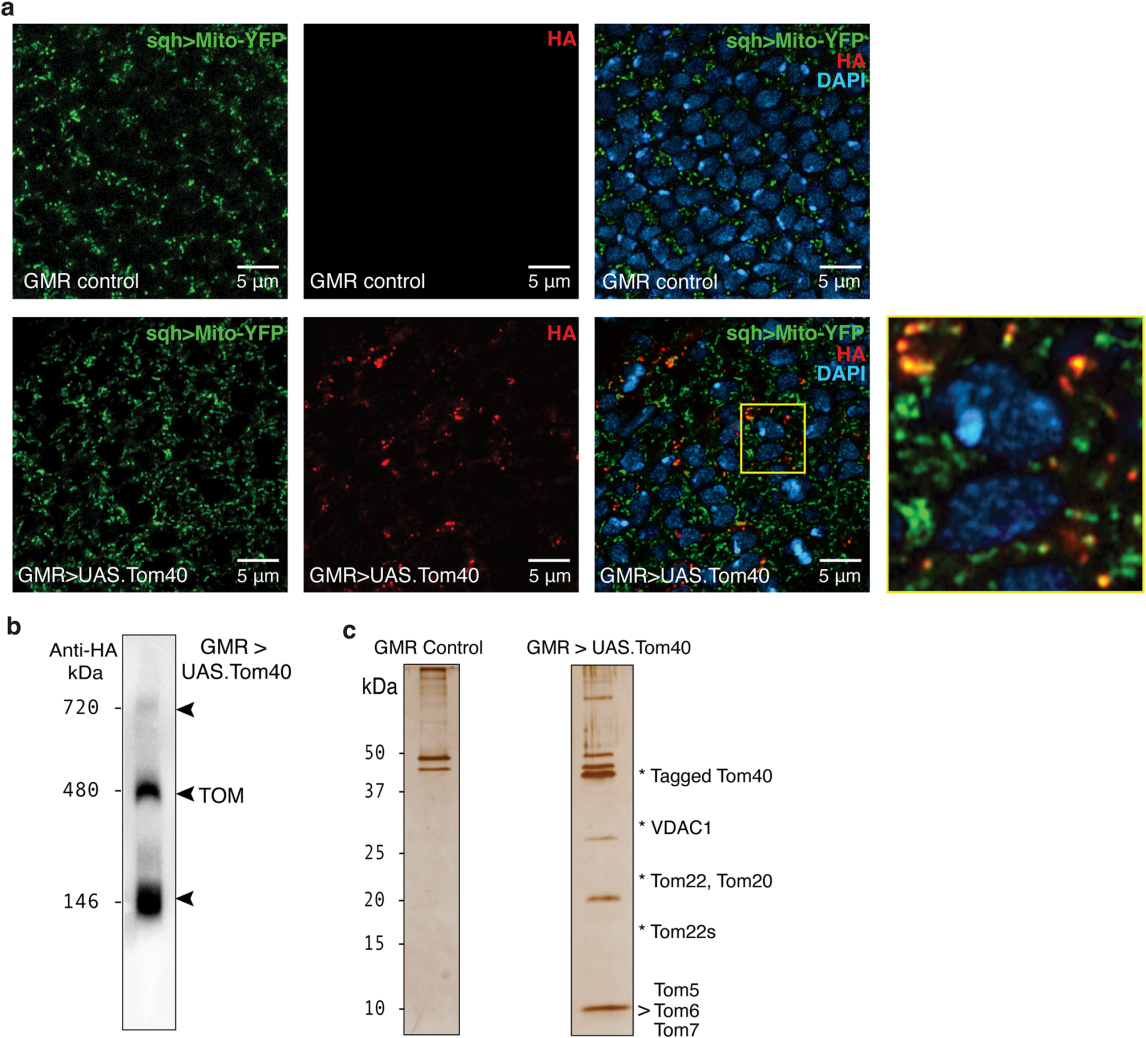
Subcellular localisation and assembly of Tom40. (**a**) Sub-cellular localisation of *UAS.Tom40*. All panels show imaginal eye discs from third instar *Drosophila* larvae expressing mitochondrially-targeted YFP (sqh > mito-YFP; green). The upper panels show sections from GMR-GAL4 (driver-only) control flies, while the lower panels are from flies co-expressing GMR-GAL4 > *UAS.Tom40* (HA antibody; red). An enlargement of the boxed area, in flies expressing *UAS.Tom40*, is shown immediately to the right. (**b**) BN-PAGE of membranes from GMR-driven Tom40-expressing eyes. Western blot analysis against a HA epitope tag reveals that Tom40 assembles into a higher order complex of nominal mass ~ 480 kDa. Additional bands migrating close to 146 kDa and 720 kDa markers indicate the presence of other Tom40-containing complexes. (**c**) Silver-stained SDS-PAGE showing affinity-purified (anti-FLAG) Tom40 samples from eye membranes solubilised with digitonin. Each sample corresponds to protein derived from ~ 800 fly heads and is representative of at least three independent samples. Affinity purified products from flies reared at 25 °C, carrying GMR only (non-specific binding control) or GMR > *UAS.Tom40*.

We monitored subcellular distribution of Tom40 in the differentiating compartment of the larval eye, demonstrating that overexpressed Tom40 protein is efficiently imported to mitochondria using fly lines co-expressing GMR-GAL4 > *UAS.Tom40* and mito-YFP (sqh > Cox8-YFP) (Fig. 3a). Differences in expression pattern under the GMR-GAL4 driver and endogenous sqh promoters^34^ may explain small inconsistencies in mito-YFP to HA overlap.

To determine whether transgenic Tom40 incorporates with endogenous *Drosophila* TOM components into discrete higher order complexes, we analysed the membrane fractions from adult fly heads. Digitonin-solubilised Tom40 migrated in three major bands, representative of assembly intermediates, TOM, and higher order assemblies, as reported^35^ (Fig. 3b, and intact western blots in Suppl. Fig. S3a), whereas fractions from HA-tagged Tom22 (GMR > *UAS.Tom22*) fly heads only co-migrated with one of these three bands (~ 480 kDa), validating it as the *Drosophila* TOM complex (Suppl. Fig. S4a; intact western blot in Fig. S4c). Several endogenous proteins co-elute with affinity-purified Tom40 (Fig. 3c; intact SDS-PAGE gels in Suppl. Fig. S3b) or Tom22 (Suppl. Fig. S4b, S4d-e). Tryptic-digest mass spectrometry of excised bands identified other TOM components including Tom22 (both normal and a short isoform), Tom20, Tom7, Q6IGW6 (previously unidentified *Dm*Tom6), Q8IRD0 (previously unidentified *Dm*Tom5), and also VDAC1.

### Eye degeneration is associated with increased assembly of TOM

As *Drosophila* carrying a single copy of *UAS.Tom40* reared at 29 °C exhibit comparable Tom40 mRNA levels and phenotype to 2x*UAS.Tom40* flies reared at a growth temperature of 25 °C, the condition 1x*UAS.Tom40* at 29 °C was utilised as a common basis for further genetic modifier screens. Genetic progeny used for phenotype analysis were thus reared at 29 °C and carry either GMR > Tom40 or GMR > with an additional modifier.

**Figure 4:**
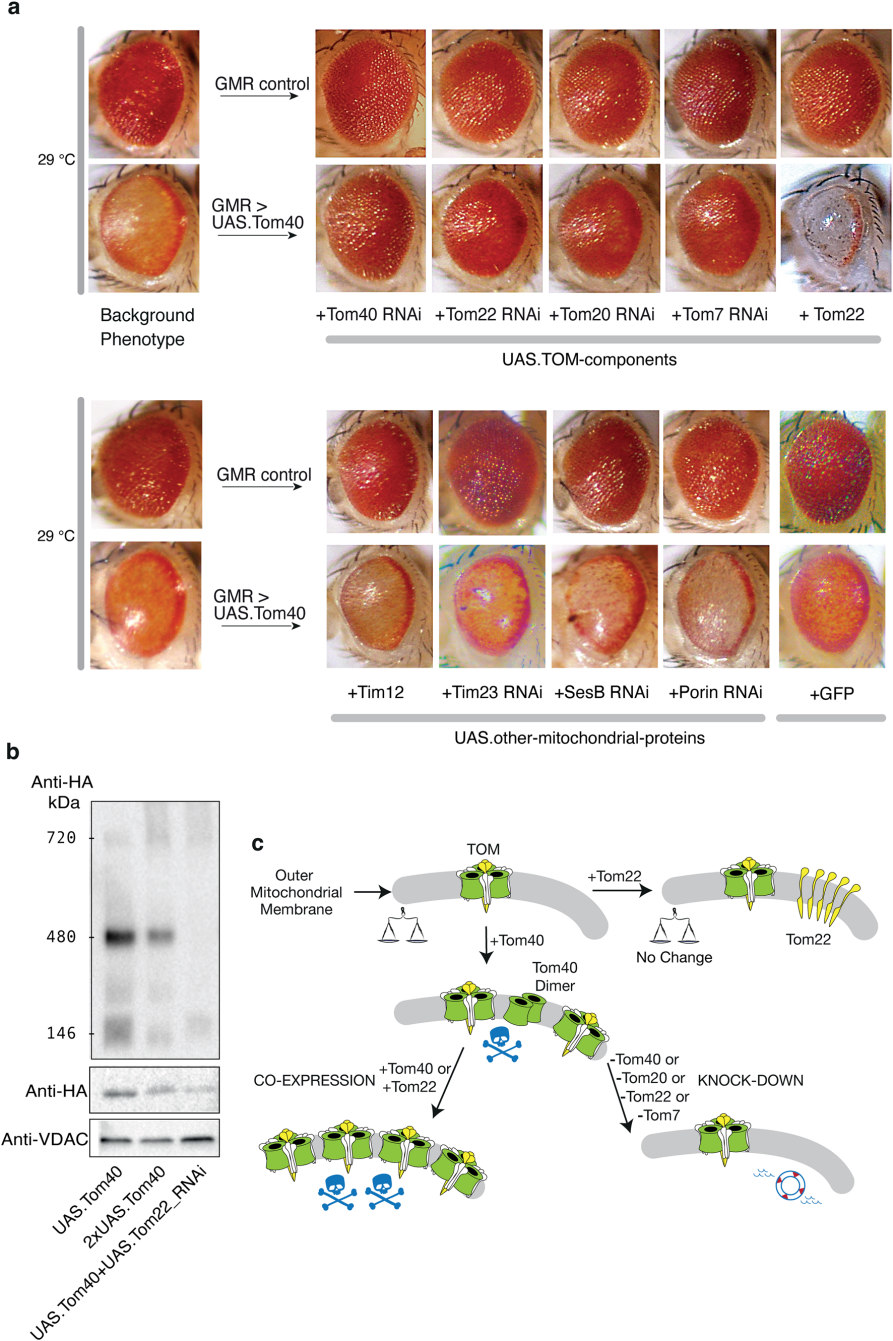
TOM components modify the Tom40 phenotype. (**a**) Phenotypes of expressing genetic modifiers in flies reared at 29 °C. Top row: Representative eye images for GMR-driven expression of UAS transgenes alone. Bottom row: Representative eye images of GMR-GAL4 > *UAS.Tom40* + UAS transgenes as labelled. For each genotype, 8–10 flies were imaged using light microscopy. (**b)** BN-PAGE analysis of Tom40 assembly in fly genotypes as labelled, showing major bands as in Fig. 2B. Below: SDS-PAGE of the same samples using VDAC (mitochondrial porin) as a mitochondrial marker, and anti-HA to monitor Tom40. Note that when 2 copies of Tom40 are expressed, a dearth of surviving eye tissue decreases the final amount of TOM for a given number of flies. (**c**) Interpretive schematic based on the relationship between genotype and eye phenotype in genotype screens on flies reared at 29 °C. Tom40 is depicted in green, Tom22 in yellow, and the other Tom subunits in outline. Normal eye phenotype is denoted by balanced scales, dying eye tissue by a skull-and-crossbones (1 = death; 2 = megadeath), and rescue (full or partial) by a lifebuoy.

Unlike Tom40, ectopic overexpression of the core TOM component Tom22 did not result in an eye phenotype (Fig. 4a). However, Tom22 co-expression strongly enhanced the Tom40 eye degeneration phenotype, resulting in eyes of a similar ablated appearance to those observed in flies overexpressing two copies of *UAS.Tom40* (Fig. 1b). A green fluorescent protein (*UAS.GFP*) overexpression control verified the phenotype was due to co-expression of the Tom22 and Tom40 subunits and not by ‘dilution’ of the driver by genetic modifiers or by overload of ectopically expressed proteins.

To explore this further, we depleted endogenous TOM components using validated RNAi lines (Suppl. Fig. S5; Table S1). Indeed, in eyes where endogenous Tom22 was knocked down and Tom40 over-expressed, the degenerative phenotype was suppressed. BN-PAGE of cell membranes from these fly heads showed depletion of the characteristic TOM band, verifying that TOM assembly was compromised, thus linking TOM assembly to phenotype (Fig. 4b; uncropped western blots are in Suppl. Fig. S6a-b). Overall depletion of ectopic Tom40 protein in flies co-expressing Tom40 and Tom22_RNAi (lane 3) relative to flies with only Tom40 expressed (lane 1), may indicate post-transcriptional co-regulation of the levels of Tom40 and Tom22.

The eye phenotype was similarly suppressed by co-knockdown of other TOM subunits, including Tom40 itself, Tom20, and Tom7 (Fig. 4a). As controls, we tested other mitochondrial proteins that, while dependent on TOM for their import to mitochondria, are not part of the TOM assembly and thus would not be expected to modify the eye phenotype if degeneration was solely due to an overabundance of TOM rather than to disruption of import. The phenotype was not modified by co-knockdown of the voltage dependent anion channel 1 (VDAC1/Porin), which is located in the outer mitochondrial membrane^36^, the adenine nucleotide transporter (ANT/SesB), or Tim23, the core component of the TIM23 translocase of the inner mitochondrial membrane^37^ (Fig. 4a). Co-expressing Tom40 with the mitochondrial inner membrane translocase (TIM22) component Tim12^38,39^ (Fig. 4a) neither enhanced nor suppressed the phenotype.

The data show that phenotype is only significantly affected by the level of TOM components, indicating that an increased load of assembled TOM triggers caspase-driven apoptotic degeneration of photoreceptor cells (Fig. 4c), in a manner that does not appear to relate directly to protein import.

### The redox-active α-crystallin Hsp22 co-assembles with Tom40 in dying eyes

**Figure 5:**
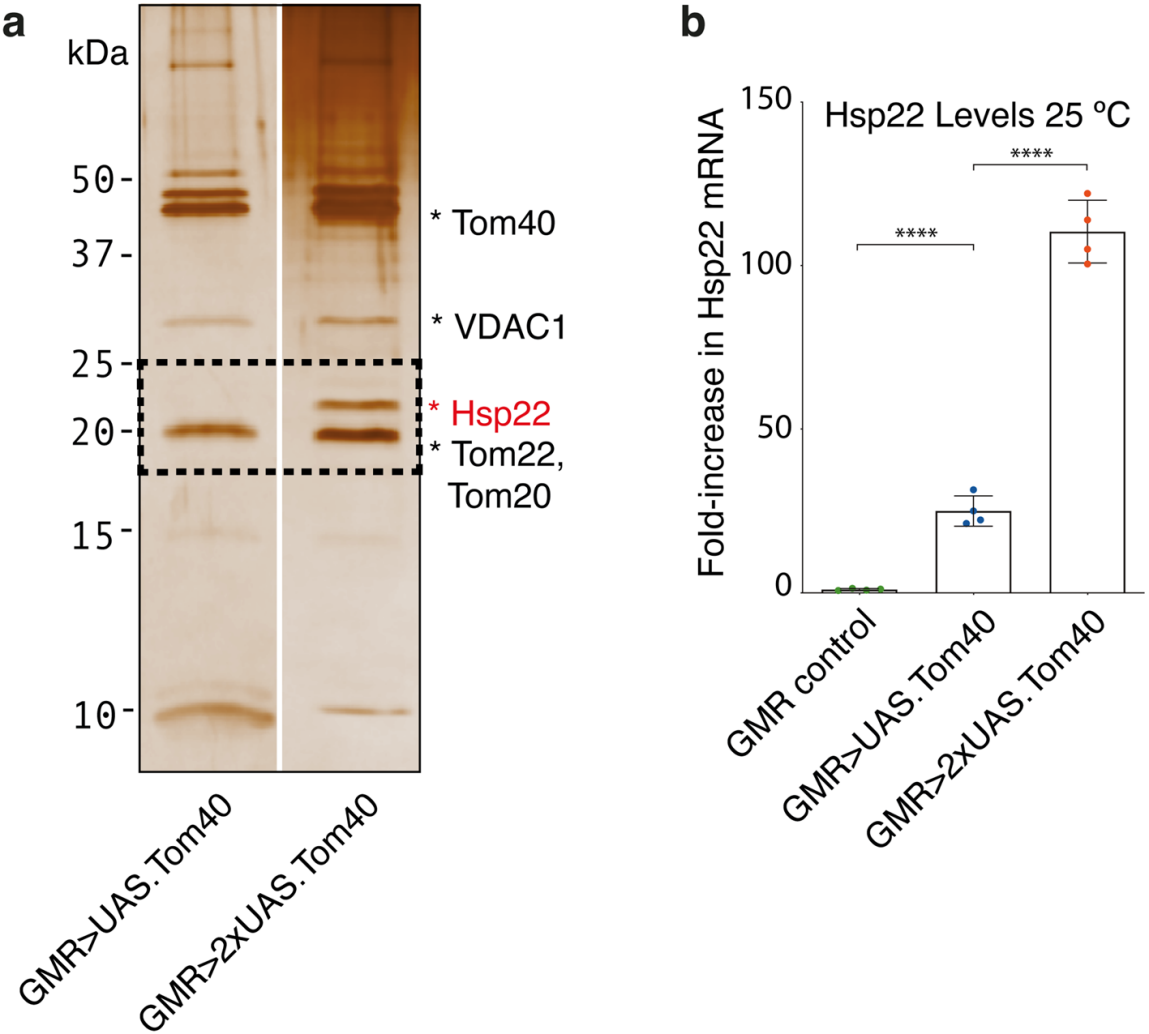
*Dm*Hsp22 is upregulated in a dose-dependent manner and associates with Tom40. (**a**) Silver-stained 15% SDS-PAGE showing affinity-purified (anti-FLAG) Tom40 samples from eye membranes solubilised with digitonin. Each sample corresponds to protein derived from ~ 800 fly heads and is representative of at least 3 independent samples. Affinity purified products from flies reared at 25 °C, carrying one (lane 1) or two (lane 2) copies of the *UAS.Tom40* transgene. The boxed area highlights Hsp22. (**b**) Quantification of *Dm*Hsp22 mRNA in flies expressing 0, 1 or 2 copies of *UAS.Tom40* at 25 °C.

Co-immunoprecipitation experiments, using FLAG-tagged Tom40 as bait, revealed an unexpected association between Tom40 and a small ATP-independent heat shock protein *Dm*Hsp22 in degenerating eyes (Fig. 5a; uncropped in Suppl. Fig. S7). The relative amount of *Dm*Hsp22 (Suppl. Fig. S8a) co-purifying with Tom40 increased with phenotype severity and Tom40 dose. *Dm*Hsp22 is upregulated under oxidative stress^40^ and localises to mitochondria^41^ via a putative N-terminal matrix-targeting sequence^41^. Reported interactions with Hsp60 (mitochondrial matrix), the F1-ATPase, other inner membrane proteins and Tom22^42^ suggest its mitochondrial distribution is likely to include the matrix, intermembrane space and inner membrane. Tom40-Hsp22 is predominantly associated with the membrane fraction of lysates (Suppl. Fig. S8b).

Negligible *Dm*Hsp22 mRNA present in the heads of 3-day old adult wild type flies is consistent with published observations that *Dm*Hsp22 protein is undetectable in heads of young flies cultured at 29 °C at 6 days, increasing with age to a maximum at ~ 35 days^43^. However, *Dm*Hsp22 mRNA levels increased substantially (> 20-fold or > 100-fold), in three-day old flies ectopically expressing one or two copies of *UAS.Tom40*, respectively (Fig. 5b), indicating specific upregulation in response to Tom40 overexpression. For comparison, we checked the mRNA levels of two classical ATP-dependent heat shock chaperones involved in protein folding and disaggregation, Hsp60 (mitochondrial matrix) and Hsc70 (Hsp70; cytosolic) in flies expressing one or two copies of *UAS.Tom40* and found no significant change from wild type flies (Suppl. Fig. S8c), suggesting *Dm*Hsp22 plays a more targeted role.

## Discussion

Here we provide evidence that elevated mitochondrial TOM levels trigger cell death in an in vivo* Drosophila* model, illuminating a possible connection to *TOMM40*-related neurodegeneration and cognitive diseases associated with ageing. While research into Alzheimer’s disease has historically focused on the linked *APOE* haplotypes, *APOE*-independent associations between poly-deoxythymidine (poly-T) variants of *TOMM40* rs10524523 (‘523’) and cognitive decline have been documented^44^. Fly retinas overexpressing Tom40 display an increase in Tom40 mRNA levels comparable to the elevated *TOMM40* mRNA detected in brain tissue from Alzheimer’s patients^45^, demonstrating the applicability of the *Drosophila* disease model. Moreover, an *APOE-TOMM40* linkage disequilibrium is not a confounding factor *in Drosophila*.

Cellular studies have shown variable effects of Tom40 expression on mitochondria. For example, in HeLa cell lines, a moderate increase in *TOMM40* expression was implicated in increased endogenous ATP and respiration^46^, while in a study using primary rat cardiomyocytes, a similar increase in *TOMM40* transcription led to upregulation of caspases 3 and 8, a diminished membrane potential, and apoptosis. The effects on cardiomyocytes, similarly to the fly eye phenotypes presented here, were abolished by expression of Tom40 RNAi^47^. Our in vivo study establishes that in the neural context of *Drosophila* retina, upregulation of Tom40 is sufficient to activate cell death and degeneration, contingent upon the availability of co-assembling TOM components.

Our data show that death of retinal neurons increases with Tom40 dose, while the accompanying activation of executioner caspases signifies apoptotic pathways are switched on. Phenotype suppression by inhibitors of either initiator or executioner caspases verifies the caspase-dependency of retinal death. Apoptosis in this context does not appear to occur by the intrinsic apoptotic pathway, suggesting excessive TOM could trigger cell death via the death receptor pathway. Moreover, we observed a correlation between the level of caspase activation and the heightening of expression of endogenous *Dm*Hsp22, a key marker of lifespan and age-related cognition in *Drosophila*^48^.

While we have focussed on Tom40 overexpression, reduction of Tom40 is also disruptive to eye integrity in the longer term, albeit due to autophagy. A study of GMR > *Tom40_RNAi* in *Drosophila* revealed normal eyes in 2-day old Tom40 knockdown flies, consistent with our 3-day data, but ommatidial degeneration due to formation of ubiquitinated aggregates and autophagy became evident in 30-day old flies^49^. This suggests that Tom40 dysregulation may act diversely as a switch in mitochondrial quality control pathways.

Mitochondrial dysfunction is an early event in neurodegeneration^50^ and Alzheimer’s disease^51^, and previous findings of TOM-driven import of pathogenic polypeptides, such as amyloid β^52^ and a-synuclein^53^ have led to speculation that elevated TOM increases import of these polypeptides, or mitochondrial import in general, which may be a factor in Alzheimer’s and other neurodegenerative diseases. While our genetic data implicate an oversufficiency of TOM in triggering neuronal death, suppression of the Tom40 phenotype is not achieved by knockdown of Tim23, which accepts precursor proteins from TOM and mediates their translocation across the intermembrane space to the mitochondrial matrix. The Tom40 phenotype similarly remains unchanged when Tim12, a component of the inner membrane translocase for inner membrane targeted precursors TIM22, is co-expressed with Tom40. It therefore seems likely that something other than excessive import of matrix or inner membrane directed protein precursors is responsible for initiating neurodegeneration.

Mitochondrial dysfunction associated with excess TOM may contribute to stress-related dysregulation of specific cytoprotective pathways. Some cell survival pathways utilize TOM for specialised recruitment or sentinel functions. For instance, Tom40 and Tom7 are essential facilitators of PINK1-PARKIN mitophagy^6,54^, in a process driven by reactive oxygen species (ROS)^55^, while Tom40 and Tom70 facilitate recruitment of the autophagy-related gene product Atg2A to the mitochondria-associated endoplasmic reticulum prior to autophagy^7^. In our study, the upregulation of *Dm*Hsp22 upon Tom40 over-expression is consistent with the cell undergoing stress, and its presence in three-day old flies signals initiation of cytoprotective responses. *Dm*Hsp22 is known to be activated in response to hypoxia and its expression can be artificially induced by low doses of paraquat, which promotes the release of ROS^56^. *Dm*Hsp22 has also been implicated in the mitochondrial unfolded protein (stress) response^56,57^. Flies with knockdown of *Dm*Hsp22 are sensitised to stress and show a 40%^40^ or more^48^ decrease in lifespan, whereas flies overexpressing *Dm*Hsp22 ectopically in neurons (specifically) have increased lifespan and better maintenance of cognition with age^48^. The neuronal link may be key, as differentiated neurons do not rejuvenate by mitosis, and a mechanism to slow their demise after the commencement of apoptosis, or to preclude apoptosis of some of the neurons present, would potentially be beneficial for the organism. Intriguingly, the closest human homologue of *Dm*Hsp22 by sequence alignment, α-crystallin B, is a regulator of intrinsic apoptosis upregulated in neurodegenerative and retinal diseases; it is neuroprotective, directly inhibiting caspase-3 activation by preventing autoproteolytic cleavage of pro-caspase-3^58^. While α-crystallin B has a broad subcellular distribution^59,60^, it has functional parallels with *Dm*Hsp22, including upregulation as ROS increase^61^, and inhibition of oxidant-driven apoptosis^62^. Whether *Dm*Hsp22 directly antagonises DCP-1 activity in this manner remains to be explored, but the distribution of DCP-1 in mitochondria as well as the cytosol^63^ could be regarded as suggestive.

## Conclusions

Findings presented here advance our knowledge of mitochondrial dysfunction in human neurodegenerative disease, revealing an unanticipated role for the TOM translocase in triggering apoptosis and neurodegeneration and a plausible rationale for the predisposition of individuals carrying intronic variants that increase expression of the *TOMM40* gene to cognitive disease. A mitochondrial cytoprotective factor (*Dm*Hsp22) that improves cognition and longevity in flies is upregulated along with Tom40, and an avenue of particular interest now would be to determine whether human *Dm*Hsp22 homologues are upregulated and associate with TOM in the brains of Alzheimer’s patients, and whether their cytoprotective functions slow disease progression.

## Materials and methods

### Experimental model

*Drosophila melanogaster* stocks cultivated on standard media were reared at 25 °C or 29 °C, as specified. All strains used in this study are detailed in Table S2.

The *UAS.Tim12-1D4* transgenic line was established as follows. *Drosophila* Tim12 DNA construct was designed with a 1D4 epitope tag, purchased as a genomic block (Integrated DNA Technologies) and cloned into pUASTattb vector (a gift from Dr Michael Murray, The University of Melbourne) between *EcoRI* and *XhoI* restriction sites. The transgenic fly line was generated (BestGene) by microinjecting 20 μg of sequence-verified plasmid into *Drosophila* embryos for site-specific chromosome recombination using the Phi31 integrase system^64^.

### General fly handling procedures

Strains were reared on molasses-based Drosophila medium. Briefly, 1.4 kg of molasses is mixed in 3 l of water and 900 g of yeast mixed with 3 l of water before pouring into a 30 l metal pot and placing on a gas burner. The mixture was brought to 100 °C with occasional stirring. 630 g of semolina was mixed with 3 l of water and added. Upon the mixture obtaining a homogeneous consistency, it was cooled to 80 °C prior to the addition of 138 ml Acid Mix (546 ml H_2_O, 412 ml Propionic acid and 42 ml Phosphoric acid) and 262 ml Tegosept (1000 ml 95% ethanol and 100 g hydrobenzoic acid methyl ester). The mixture was dispensed into trays of polypropylene Drosophila culture vials using the "drosofiller" automated fly food dispenser (Flystuff, CA).

### Genetic crosses

Male and unmated female (virgin) flies from two different genotypes were crossed. To select flies for gender, virginity and phenotypic markers, freshly eclosed flies were tipped from their vial onto a porous pad dispensing CO_2_ connected to a dissection light microscope for observation.

For modifier screens, a stable homozygous GMR > *UAS.Tom40* line was generated and used as one parent in each cross with fly lines containing modifier genes. Thus, all progeny from the crosses contain one copy of GMR > *UAS.Tom40* and one copy of the modifier gene.

### Fine dissection of Drosophila eye discs

Larval mouth hooks were clasped with forceps. Using a second pair of forceps the two brain lobes were removed by closing the second forceps in the space between the brain and eye discs and gently pulling the brain away from the mouth hooks. While continuing to hold onto the mouth hooks, the tissue was pinched off as close as possible to the connection between the antennal section of the eye-antennal disc and the mouth hooks, ensuring the eye-antennal disc complexes were free of all tissue. Eye-antennal discs were moved, one at a time, and added to the 10ul of 80% glycerol on a new slide, separated and spread out. A coverslip was placed onto the specimen. Slides were stored at 4 °C until ready to view the eye-antennal using fluorescent microscopy.

### Immunostaining, confocal microscopy and image analysis

The heads of third instar larvae were dissected in phosphate-buffered saline (PBS; Sigma Aldrich) and fixed in 4% para-formaldehyde, washed in PBS with 0.1% Triton-X (PBT) and blocked in 5 mg ml^−1^ Blocking Solution (Goat Serum in PBT; Sigma), followed by primary antibody incubation overnight at 4 °C. After washing in PBT, larval heads were incubated with fluorescently-tagged secondary antibodies. Subsequently, tissues were incubated in DAPI solution (Sigma; 1′ 4′ 6-Diamidino-2-Phenylindole diluted 1:1000 in distilled water) and extensively washed in PBS. Tissues were stored in 80% glycerol in PBT at 4 °C prior to dissection. All antibodies were diluted in Blocking Solution as above. Eye imaginal discs were dissected under an Olympus SZ microscope and mounted in 80% glycerol on a glass microscope slide, placed under a glass coverslip and sealed with clear nail polish.

A Zeiss LSM 800 running Zen Blue software was used to visualize and analyse the eye imaginal discs. Serial Z-series were captured in 1 μm sections at 40 × magnification. Fluorophores were imaged using band-pass filters to remove cross-detection between channels and pseudo-coloured for image preparation. Confocal images in generic (.czi) format were processed using Image J 1.48v, and Adobe Photoshop CS5 Version 12.0.4.I. For caspase pixel intensity quantification (quantification of DCP-1 staining), images were imported into Imaris software and the caspase signal set to basal level. A 3D volume-based measurement of caspase and DAPI signals in the posterior region of the eye discs was carried out. Proportional caspase staining (to DAPI) was calculated, and graphs plotted in Prism 8.3.1.

Tom40-HA levels were quantified using FIJI (version 2.1.0/1.53c). Data points represent different eye discs with the relevant genotype. For each image, 3 μm sections were merged with maximum projection, 5 ommatidia per image were selected based on DAPI staining to identify nuclei, and the average raw Tom40-HA intensity was measured within the selection. For quantification of Mito-YFP area proportion, 5 μm sections were merged and Auto Local Threshold (Bernsen method) applied to calculate the proportion of total area. Different eye discs were used to generate each data point.

Adult flies were anesthetized under CO_2_ under an Olympus SZ microscope Dissecting Microscope, with a KL 1500 LCD light source (Carl Zeiss) and imaged at the highest possible magnification.

### Quantitative PCR

For Tom40 expression or RNAi analyses, RNA was isolated from equivalent numbers of adult or larval heads (10 for each genotype) respectively. For RNAi line validation, Tsgal80; tubulin GAL4 system^65^ was used. RNA purity and integrity were assessed using an automated electrophoresis system (2200 TapeStation, Agilent Technologies). Each cDNA synthesis used 6 ml of RNA was used for (GoScript™ Reverse Transcription System kit, Promega). qPCR was performed using Fast SYBR Green Master Mix (Applied Biosystems) using the StepOnePlus Real-Time PCR System and Sequence Detection Systems in 96-well plates (Applied Biosystems, 95 °C for 2 min, 40 cycles 95 °C 1 s and 60 °C 20 s). Amplicon specificity was verified by melt curve analysis. Average Ct values for two technical replicates were calculated for each sample. Multiple internal control genes were analyzed for stability and target gene expression was normalized to the mean of Cyp1 and tubulin, selected for having high expression and little sample-to-sample variability as determined by RefFinder. Fold change was determined using the 2-∆∆CT method. Primers used for the experiments are provided in Table S3.

### Isolation of protein complexes

Fly heads were isolated by briefly vortexing vitrified flies with 3.5 mm glass beads (BioSpec) pre-cooled in liquid nitrogen and fractionated using four stacked pre-chilled stainless-steel sieves of decreasing pore diameter (Impact Test Equipment Ltd, UK; Mesh sizes: 710, 500, 355 and 250 mm), with vigorous manual agitation. Fly heads collected in the 500 mm sieve were homogenised using a Dounce tissue grinder in Lysis Buffer (20 mM Tris–HCl pH 7.4, 150 mM NaCl) supplemented with DNAse I (Merck Millipore) and protease inhibitor cocktail (Roche). Crude lysates were filtered using a 100 μm filter unit to remove debris and ultracentrifuged at 100,000 × *g* for 1 h at 4 °C. The pellet containing the cellular membrane fraction was retained.

Membrane pellets were resuspended in Lysis Buffer supplemented with 1% (w/v) high purity, low turbidity (< 2.5 NTU) water-soluble digitonin (Carbosynth # D-3200) and incubated at 4 °C for 30 min. Samples were centrifuged at 20, 000 × *g* for 30 min at 4 °C to remove non-solubilised material. Filtered supernatants were applied to M2 FLAG antibody-conjugated beads (ANTI-FLAG® M2 Affinity Gel; Sigma-Aldrich A2220) preequilibrated in Lysis Buffer containing 0.1% digitonin (Wash Buffer), and after extensive washing followed by Wash Buffer containing 1 M urea, and subsequently 500 mM NaCl, and re-equilibrated in Wash Buffer prior to elution using FLAG peptide (DYKDDDDK; Sigma-Aldrich F3290) at 0.5 mg ml^−1^. The elution step was repeated and the eluates pooled and concentrated using a 100 kDa centrifugal filter (Merck-Millipore).

### Protein detection, western blotting and immunodetection

SDS-PAGE used 15% polyacrylamide Tris–Glycine gels and dual colour Precision Plus Prestained Protein Standards (5 μl). Proteins were visualised by silver staining. Tryptic digests of protein bands were analysed using nano-liquid chromatography coupled with electrospray ionization mass spectrometry (nano-LC-ESI MS/MS). Mass spectra were recorded on a QExactive Plus 1 machine at the Monash Proteomics Facility. Data analysed using Mascot software V2.4 (Matrix Science) were searched against the protein sequence databases Uniprot and Swissprot.

BN-PAGE analysis of solubilised cell membranes from adult fly heads was performed at 4 °C using the Native PAGE Bis–Tris Gel System (Invitrogen) on 4–16% Bis–Tris gels using NativeMark unstained protein standards. Tagged Tom40 was detected by western blot against the HA peptide.

Details of antibodies are provided in Table S4. Protein bands from SDS-PAGE or BN-PAGE were transferred to methanol-activated 0.2 μm PVDF membranes (Millipore) using the semi-dry transfer method^66,67^. Gels and thick blot filter papers (Bio-Rad) were soaked in Western Transfer Buffer (25 mM Tris–HCl pH 8.3, 192 mM glycine, 10% (v/v) methanol, 0.05% (w/v) SDS) for 5 min prior to electroblotting. PVDF membranes (Merck Millipore) were incubated in Western Blocking Solution (5% (w/v) skim milk powder, 0.05% TWEEN-20, made up with PBS for 1 h at room temperature on a rolling platform. For BN-PAGE gels, the transfer membrane was de-stained with methanol before blocking. After incubation with an anti-HA monoclonal antibody (1:1000), or anti-FLAG monoclonal antibody (1:1000), the membrane was thrice washed with Western Wash Buffer (PBS + 0.05% TWEEN-20) before adding rabbit anti-rat secondary antibody, or goat anti-mouse secondary antibody, conjugated to horseradish peroxidase (1:5000). After washing, it was treated with 5 ml of Immobilon Western Chemiluminescent HRP Substrate (Merck) for 5 min. Chemiluminescent protein bands were detected using a ChemiDoc system (Bio-Rad).

### Quantification and statistical analysis

All statistical tests were performed with Graphpad Prism 8 using an unpaired two-tailed t-test with 95% confidence intervals. In all figures, error bars represent SD and according to the Graphpad classification of significance points * (P = 0.01–0.05), ** (P = 0.001–0.01), *** (P = 0.0001–0.001) and **** (P < 0.0001). N values indicate the number of biological replicates for a sample.

## Supplementary Information

Supplementary Information.

## Data Availability

All data and reagents relevant to this study are available from the corresponding authors upon reasonable request.
