# Supplementary material for: An increase in mitochondrial TOM activates apoptosis to drive retinal neurodegeneration
Source: Sci Rep. 2022 Dec 14;12:21634. doi: 10.1038/s41598-022-23280-z (PMC9750964; doi:10.1038/s41598-022-23280-z)
Supplement: Supplementary file 1 — Supplementary Information. [file 41598_2022_23280_MOESM1_ESM.pdf]

An increase in mitochondrial TOM activates apoptosis to drive retinal neurodegeneration

Agalya Periasamy, Naomi Mitchell, Olga Zaytseva, Arjun Singh Chahal, Jiamin Zhao, Peter M Colman, Leonie M Quinn and Jacqueline M Gulbis.

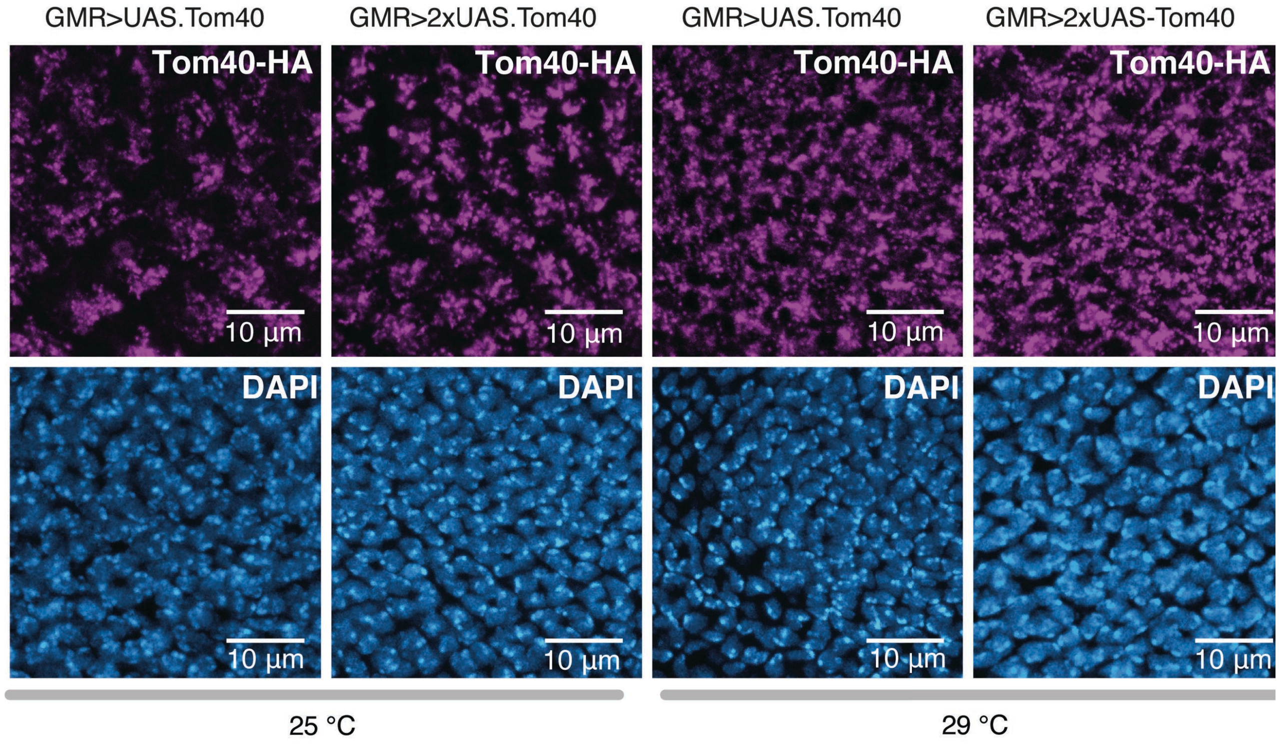

Figure S1. Increased Tom40 transcription leads to apoptosis and eye degeneration. Representative images for quantification of HA-tagged Tom40 expression (Fig. 1d) in the posterior region of eye imaginal discs. Each image is representative of three replicates.

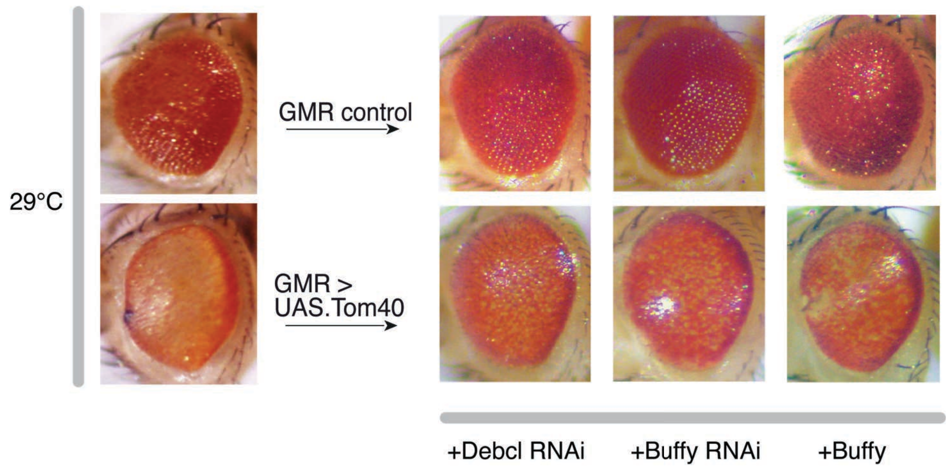

Figure S2. Apoptosis is unaffected by knockdown of BCl-2 proteins Buffy or Debcl.

Top row: Representative eye images for GMR-driven expression of UAS transgenes alone. Bottom row: Representative eye images of GMR-GAL4>*UAS.Tom40* + UAS RNAi or overexpression transgenes as labelled. +Debcl flies do not hatch and thus are not shown here. For each genotype, 8 - 10 flies were imaged under a light microscope.

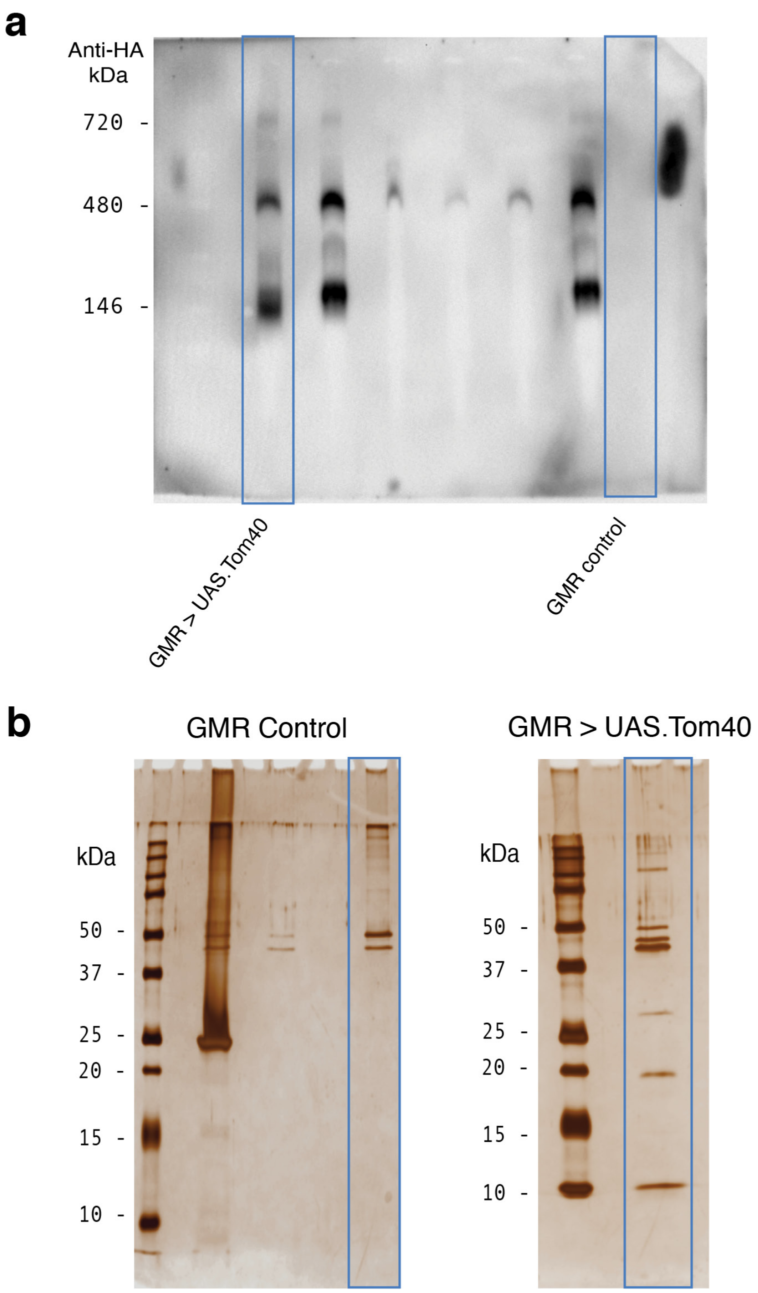

Figure S3. a. Whole gel western blot of the BN-PAGE used in Fig. 3b. **b.** Whole gel images of the silver-stained SDS-PAGE lanes used for Fig. 3c. Relevant lanes are boxed in blue.

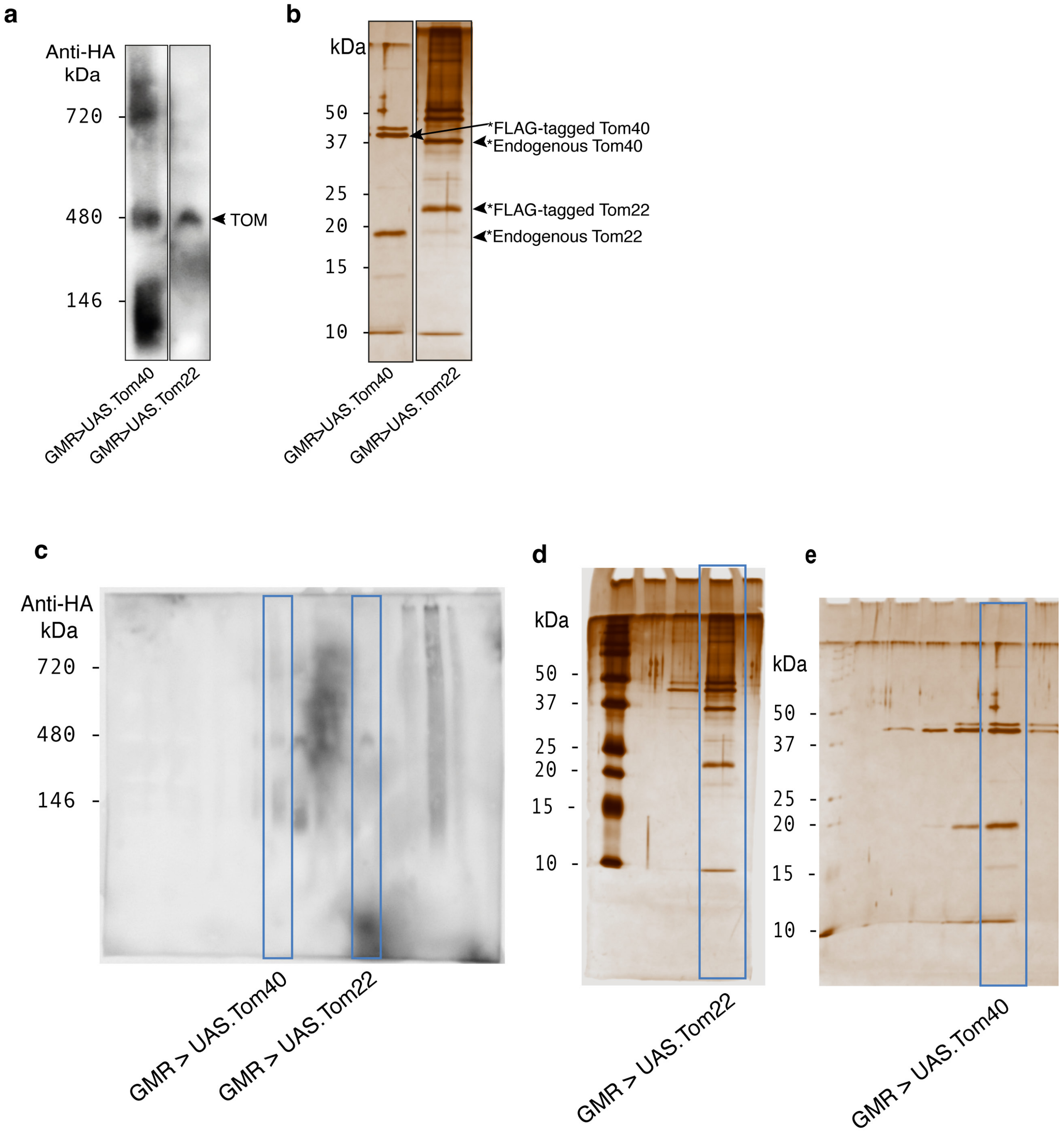

Figure S4. Ectopically expressed Tom40 and Tom22 assemble into TOM *in vivo*.

a. Solubilised membrane fractions derived from flies expressing GMR-GAL4>*UAS.Tom40* and GMR-GAL4>*UAS.Tom22* on BN-PAGE as marked. **b.** Silver-stained SDS-PAGE of affinity-purified (anti-FLAG) samples from eye membranes of GMR-GAL4>*UAS.Tom40* and GMR-GAL4>*UAS.Tom22* flies, as marked. Each sample corresponds to protein derived from ~ 800 fly heads and is representative of at least three independent samples. **c.** Original unprocessed BN-PAGE western blot of panel a. **d-e.** Intact silver-stained SDS-PAGE gels used for panel b, as marked. Relevant lanes are boxed in blue.

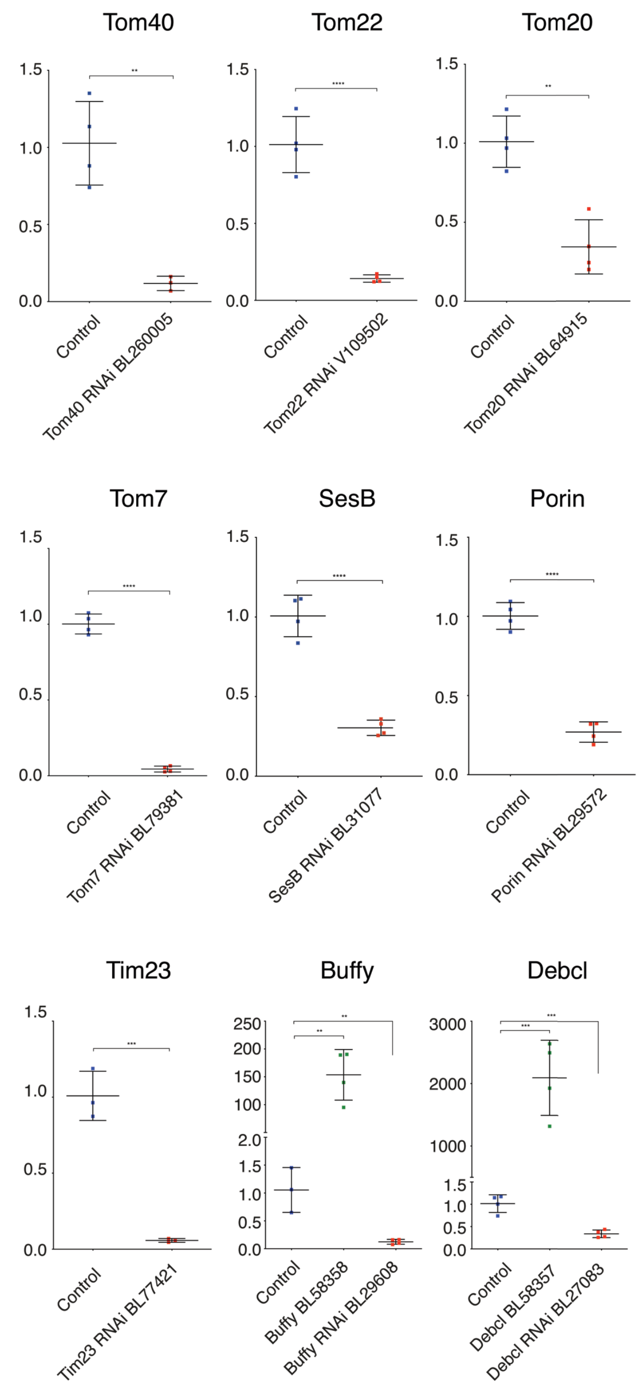

Figure S5. Candidate genetic analysis was used to determine the role of TOM assembly in triggering the eye phenotype. Knockdown/overexpression efficiency, as indicated, as assessed by qPCR for Tom40, Tom22 (*Dm*Mge1), Tom20, Tom7, VDAC1 (*Dm*Porin), ANT1 (*Dm*SesB), Tim23, Buffy (Bax homologue) and Debcl (Bcl-2 homologue) mRNA abundance in *Drosophila* third instar larval eye discs expressing the indicated dsRNAs. Expression was normalized to driver only controls. In each panel, data from at least three independent replicates are represented as mean ± SD. Asterisks indicate p-values ≤0.05 by an unpaired T-test.

a

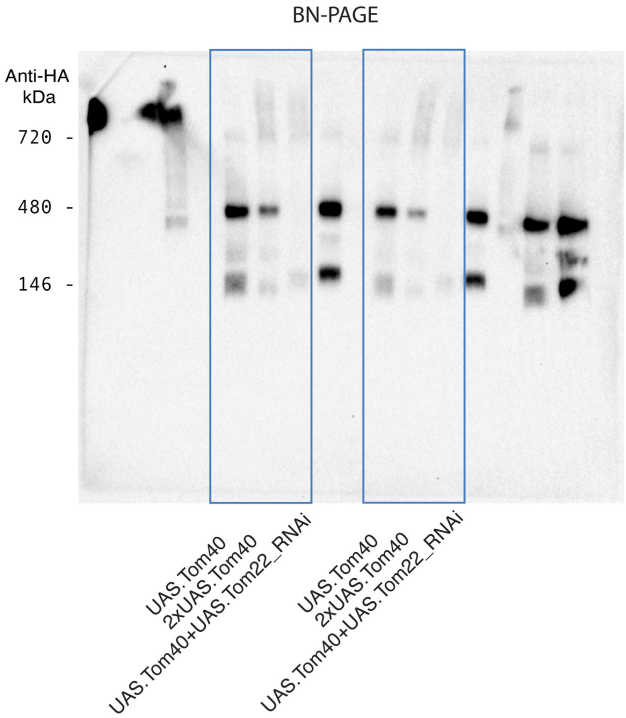

b

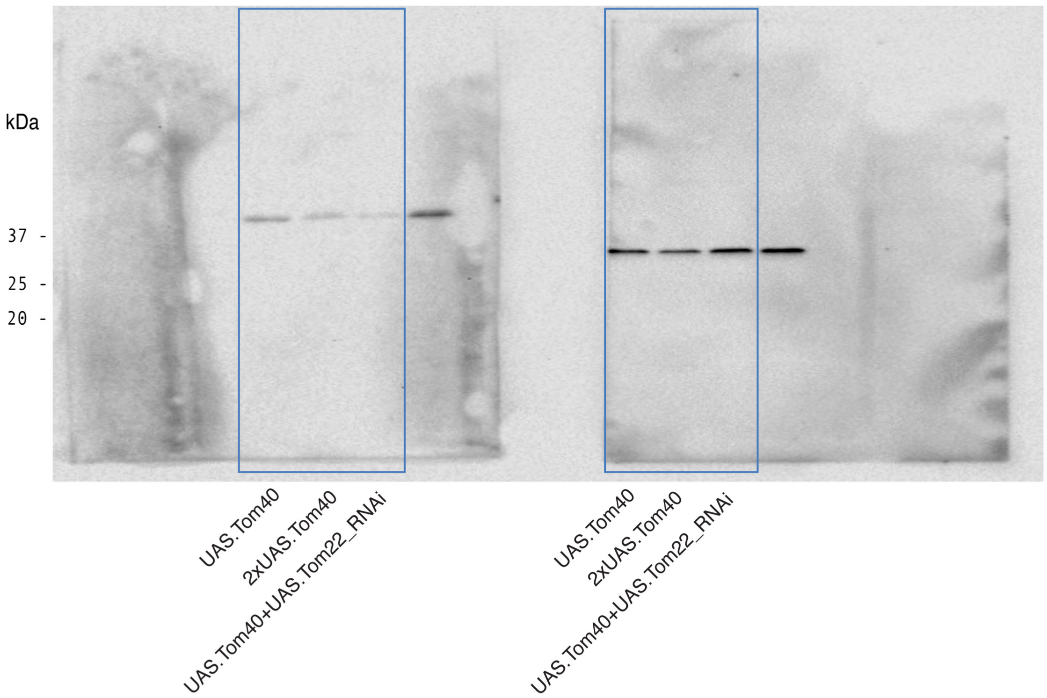

Figure S6. a. Whole (BN-PAGE) western blot image relevant to Fig. 4b. **b.** Whole (SDS-PAGE) western blot images relevant to Fig. 4b. Relevant lanes are boxed in blue.

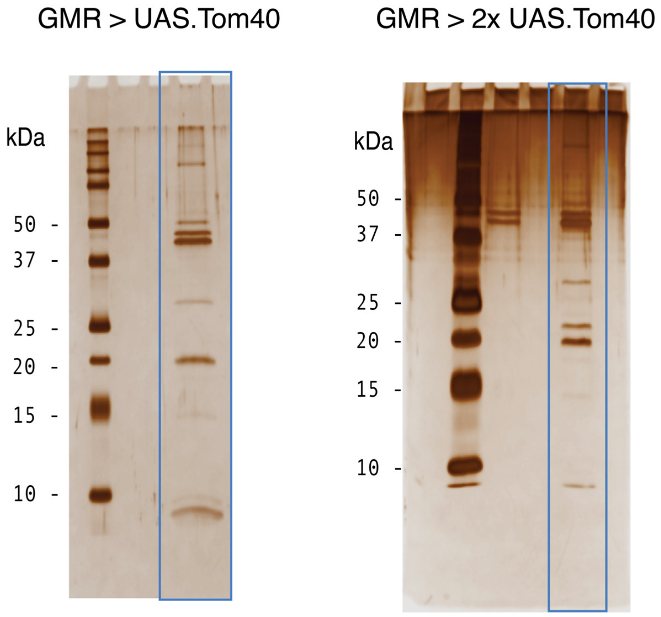

Figure S7. Whole gel images of silver-stained SDS-PAGE used in Fig. 5a. Relevant lanes are boxed in blue.

a

```
SP|P02515|HSP22_DROME --------------------MRSLPMFWRMAEEMARMPRLSSPFHAFFHEPPVWSVALPR 40
SP|P02511|CRYAB_HUMAN MDIAIHHPWIRRPFFPFHSPSRLFDQFFGEHLLESDLFPTSTSLSPFYLRPPS------- 53
                                           * :  *:      : :   *: :  *: .**

SP|P02515|HSP22_DROME NWQQIARWQEQEFAPPATVNKDGYKLTLDVKDYSEL--KVKVLDESVVLVEGKSEQQEAE 98
SP|P02511|CRYAB_HUMAN -FLRAPSWFDTGLS-EMRLEKDRFSVNLDVKHFSPEELKVKVLGDV-IEVHGKHE-ERQD 109
                       : :   * :  ::    ::** :.:.****.:*    *****.:  : *.** * :. :

SP|P02515|HSP22_DROME QGGYSSRHFLRRFVLPEGYEADKVTSTLSSDGVLTISVPNPPGVQETLKEREVTIEQTGE 158
SP|P02511|CRYAB_HUMAN EHGFISREFHRKYRIPADVDPLTITSSLSSDGVLTVNGPRKQV---SGPERTIPITREEK 166
                      : *: **.* *:: :* . :  .:**:********:. *.      :  ** : * :  :

SP|P02515|HSP22_DROME PAKKSAEEPNDKAASQ 174
SP|P02511|CRYAB_HUMAN PAVTAAPKK------- 175
                      ** .:* :
```

Identity 29.7%
Similarity 46.2%

```
SP|P02515|HSP22_DROME ----------MRSLPMFWR----------MAEEMARMPRLSSPFHAFFHEPPVWSVALPR 40
SP|P02489|CRYAA_HUMAN MDVTIQHPWFKRTLGPFYPSRLFDQFFGEGLFEYDLLPFLSSTISPYYRQS--------- 51
                                 *:*  *:              *   :* *** :  ::::

SP|P02515|HSP22_DROME NWQQIARWQEQEFAPPATVNKDGYKLTLDVKDYSELK--VKVLDESVVLVEGKSEQQEAE 98
SP|P02489|CRYAA_HUMAN ----LFRTVLDSGISEVRSDRDKFVIFLDVKHFSPEDLTVKVQDD-FVEIHGKH-NERQD 105
                          : *   :.    .  ::* : : ****.:*  .  *** *: .* :.**  ::. :

SP|P02515|HSP22_DROME QGGYSSRHFLRRFVLPEGYEADKVTSTLSSDGVLTISVPNPP-GVQETLKEREVTIEQTG 157
SP|P02489|CRYAA_HUMAN DHGYISREFHRRYRLPSNVDQSALSCSLSADGMLTFCGPKIQTGLDATHAERAIPVSREE 165
                      : ** **.* **: **.. : . ::.:**:**:**:. *:   *:: *  ** : :.:

SP|P02515|HSP22_DROME EPAKKSAEEPNDKAASQ 174
SP|P02489|CRYAA_HUMAN KPTSAPSS--------- 173
                      :*:.  :.
```

Identity 24.6%
Similarity 39.5%

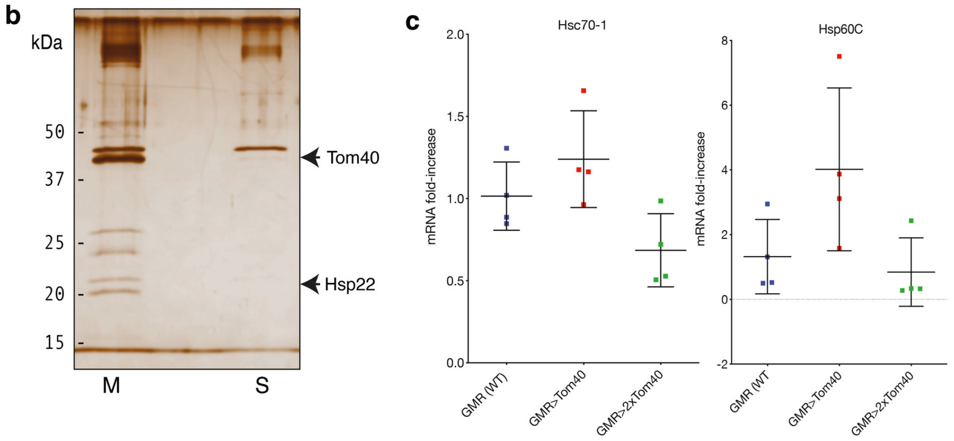

Figure S8. DmHsp22 is related to human cytoprotective chaperones and recruited to Tom40 in stressed cells. a. Pair-wise protein sequence alignments of Drosophila Hsp22 with the most similar lens-crystallin proteins found in humans: α-crystallin B (CRYAB) and α–crystallin A (CRYAA). Drosophila Hsp22 has the highest homology to Human CRYAB. Alignments were performed using the EMBOSS Needle program (EMBL EBI). **b**. Silver-stained SDS-PAGE of affinity-purified (anti-FLAG) fractions from eyes of GMR-GAL4>2 x Tom40 flies reared at 25°C shows a Tom40-Hsp22 complex is confined to the membrane fraction: Digitonin-solubilised membranes (M) and supernatant (S). Membrane and supernatant fractions were separated by centrifugation at 100,000 x g and the membranes digitonin-solubilised and applied to anti-FLAG resin. The supernatant (cytosolic) fractions were separately applied to anti-FLAG resin. Bound proteins were eluted by FLAG peptides. Hsp22 and Tom40 bands were predominantly present in eluate from membrane fraction. **c.** Quantification of Hsc70-1 and Hsp60C mRNA levels in wild type adult flies and flies expressing 1 or 2 copies of GMR-GAL4>*UAS.Tom40* raised at 25°C.

Table S1. List of TRiP RNAi fly strains used in the study

Genotype	Source	Identifier Number
UAS-Tom40 TRiP RNAi	Bloomington Drosophila Stock Center	BDSC:26005
UAS-Tom20 TRiP RNAi	Bloomington Drosophila Stock Center	BDSC:64915
UAS-Tom7 TRiP RNAi	Bloomington Drosophila Stock Center	BDSC:79381
UAS-porin TRiP RNAi - 2	Bloomington Drosophila Stock Center	BDSC:29572
UAS-sesB TRiP RNAi	Bloomington Drosophila Stock Center	BDSC:31077
UAS-Tom22 RNAi	Vienna Drosophila Resource Center	VRDC:109502
UAS-Tim23 RNAi	Bloomington Drosophila Stock Center	BDSC: 77421
UAS-Hsc70-1 RNAi	Bloomington Drosophila Stock Center	BDSC: 34527
UAS-Hsp60A RNAi	Bloomington Drosophila Stock Center	BDSC: 34729
UAS-Buffy RNAi	Bloomington Drosophila Stock Center	BDSC: 29608
UAS-Debcl RNAi	Bloomington Drosophila Stock Center	BDSC: 58357

Table S2. References for fly stocks.

D. melanogaster: UAS-Tom40-FLAG.HA	DPiM: 0631	Guruharsha, K.G., Obar, R.A., Mintseris, J., Aishwarya, K., Krishnan, R.T., VijayRaghavan, K., Artavanis-Tsakonas, S., 2012. Drosophila Protein interaction Map (DPiM) A paradigm for metazoan protein complex interactions. Fly (Austin) 6, 246–253.
D. melanogaster: UAS-Tom22-FLAG.HA	DPiM: 1045	Guruharsha, K.G., Obar, R.A., Mintseris, J., Aishwarya, K., Krishnan, R.T., VijayRaghavan, K., Artavanis-Tsakonas, S., 2012. Drosophila Protein interaction Map (DPiM) A paradigm for metazoan protein complex interactions. Fly (Austin) 6, 246–253.
D. melanogaster: GMR-GAL4 w1118; P{GMR-GAL4.w-}2/CyO	BDSC: 9146	Perrin, L., Bloyer, S., Ferraz, C., Agrawal, N., Sinha, P., Dura, J.M. (2003). The leucine zipper motif of the Drosophila AF10 homologue can inhibit PRE-mediated repression: implications for leukemogenic activity of human MLL-AF10 fusions. Mol. Cell. Biol. 23(1): 119--130.
D. melanogaster: eYFP-mito w[*]; P{w[+mC]=sqh-EYFP-Mito}3	BDSC: 7194	LaJeunesse DR, Buckner SM, Lake J, Na C, Pirt A, Fromson K. Three new Drosophila markers of intracellular membranes. Biotechniques. 2004 May;36(5):784-8, 790.
D. melanogaster: UAS-DIAP1 w[*]; P{w[+mC]=UAS-DIAP1.H}3	BDSC: 6657	Jiang, C., Baehrecke, E.H., Thummel, C.S. (1997). Steroid regulated programmed cell death during Drosophila metamorphosis. Development 124(22): 4673--4683.
D. melanogaster: UAS-P35 w[*]; P{w[+mC]=UAS-p35.H}BH2	BDSC: 5073	Jiang, C., Baehrecke, E.H., Thummel, C.S. (1997). Steroid regulated programmed cell death during Drosophila metamorphosis. Development 124(22): 4673--4683.
D. melanogaster: UAS-Buffy w[*]; P{w[+mC]=UAS-Buffy.Q}2	BDSC: 58358	Quinn, L., Coombe, M., Mills, K., Daish, T., Colussi, P., Kumar, S., Richardson, H. (2003). Buffy, a Drosophila Bcl-2 protein, has anti-apoptotic and cell cycle inhibitory functions. EMBO J. 22(14): 3568--3579.
D. melanogaster: UAS-Debcl	BDSC: 58357	Quinn, L., Coombe, M., Mills, K., Daish, T., Colussi, P., Kumar, S., Richardson, H. (2003). Buffy, a Drosophila Bcl-2 protein, has anti-apoptotic and cell cycle inhibitory functions. EMBO J. 22(14): 3568--3579.
D. melanogaster: tubulin-GAL4 y[1] w[*]; P{w[+mC]=tubP-GAL4}LL7/TM3, Sb[1] Ser[1]	BDSC: 5138	Luo, L., Lee, T., Nardine, T., Null, B., Reuter, J. (1999). Using the MARCM system to positively mark mosaic clones in Drosophila. D. I. S. 82(): 102--105.
D. melanogaster: tubulin-GAL80ts w[*]; P{w[+mC]=tubP-GAL80[ts]}20; TM2/TM6B, Tb[1]	BDSC: 7019	McGuire, S.E., Le, P.T., Osborn, A.J., Matsumoto, K., Davis, R.L. (2003). Spatiotemporal rescue of memory dysfunction in Drosophila. Science 302(5651): 1765--1768.
D. melanogaster: w1118	BDSC: 3605	Hazelrigg, T., Levis, R., Rubin, G.M. (1984). Transformation of white locus DNA in Drosophila: Dosage compensation, zeste interaction, and position effects. Cell 36(): 469--481.
D. melanogaster: Tom40-GFP PBac{fTRG01388.sfGFP-TVPTBF}VK00033	VRDC:318357	Sarov M, Barz C, Jambor H, Hein MY, Schmied C, Suchold D, Stender B, Janosch S, K J VV, Krishnan RT, Krishnamoorthy A, Ferreira IR, Ejsmont RK, Finkl K, Hasse S, Kämpfer P, Plewka N, Vinis E, Schloissnig S, Knust E, Hartenstein V, Mann M, Ramaswami M, VijayRaghavan K, Tomancak P, Schnorrer F. A genome-wide resource for the analysis of protein localisation in Drosophila. Elife. 2016 Feb 20;5:e12068.
D. melanogaster: TriP RNAi strains, targeting TOM subunits, and others (porin and sesB)		Ni, J.Q., Liu, L.P., Binari, R., Hardy, R., Shim, H.S., Cavallaro, A., Booker, M., Pfeiffer, B.D., Markstein, M., Wang, H., Villalta, C., Laverty, T.R., Perkins, L.A., Perrimon, N. (2009). A Drosophila Resource of Transgenic RNAi Lines for Neurogenetics. Genetics 182(4): 1089--1100.
Tom22RNAi VRDC	VRDC:109502	Dietzl, G., Chen, D., Schnorrer, F. et al. A genome-wide transgenic RNAi library for conditional gene inactivation in Drosophila. Nature 448, 151–156 (2007).

Table S3. List of primers used for qPCR

Gene	Primers
Tom40 (Tomm40)	5′ – CTGCAAATTCGCTTCGCAGA – 3′<br>5′ – CGTCAGCGAGAGGGTGTAAT – 3′
Mge1 (Tom22; Tomm22)	5′ – CTCTATGAACATCCGGCCCC – 3′<br>5′ – TGAAGTTTTGCAACGGCTCG – 3′
Tom20 (Tomm20)	5′ – TCCTCCCTTCCAGCTCAAGT – 3′<br>5′ – TTTGGCTCCGTCCAGTTGTT – 3′
Tom7 (Tomm7)	5′ – TGAAGCTATCCGAGGGAGTT – 3′<br>5′ – TCCCCAGTGGAATCCAGTCT – 3′
Porin (VDAC1)	5′ – CAGTCTGGAAACAAGAACGGC – 3′<br>5′ – CATCCGAATCGGCCTTGACG – 3′
sesB (ANT)	5′ – AAGTACAAGCAGGTCTTCCTGG – 3′<br>5′ – AGTTGCCGGCGAAGTAGC – 3′
Tim23	5′ — ACAAGTCAACAGCTGGCCTT – 3′<br>5′ — ATAGAGGGACGAGATGCCCA – 3′
Hsp22	5′ — CTGACCATCAGTGTGCCCAA – 3′<br>5′ — CTGCTCGATGGTCACCTCAC – 3′
Hsc70-1	5′ — GCGTACTTCAACGACTCCCA – 3′<br>5′ — TGGTGCCCTGCTTATCCAAG – 3′
Hsp60C	5′ — AGCCAATATGGTACGGCTCG – 3′<br>5′ — TGAGCAGCATTGTGTCGTCT – 3′
Buffy	5′ — GGTGGAGATTACGTGGAGCA – 3′<br>5′ — TTGATGCCACTCCAACCACC – 3′
Debcl	5′ — GGTAGCGTACCCAATCCCTC – 3′<br>5′ — TCACCTTCCGGTTGAGGACT – 3′
Tub84B	5′ — TCAGACCTCGAAATCGTAGC – 3′<br>5′ — AGCCTGACCAACATGGATAGAG – 3′
Cyp1	5′ — TCGGCAGCGGCATTTCAGAT – 3′<br>5′ — TGCACGCTGACGAAGCTAGG – 3′

Table S4. Antibodies used in this study.

REAGENT or RESOURCE	SOURCE	IDENTIFIER
**Antibodies**		
Anti-HA, rat monoclonal	Roche	11867423001
Anti-FLAG, mouse monoclonal	Sigma Aldrich	F3165
Anti-VDAC1 / Porin, mouse monoclonal	Abcam	ab14734
Anti-Drosophila caspase-1 (DCP-1), Rabbit polyclonal	Cell Signalling Technologies	9578
Anti-rat HRP, Rabbit polyclonal	ThermoFisher Scientific	61-9520
Anti-rat rhodamine, Goat polyclonal	Invitrogen	31680
Anti-rat 647, Chicken polyclonal	Invitrogen	21472
Anti-mouse HRP, goat polyclonal	Sigma Aldrich	M8642
